# MicroRNA-146a-5p enhances T helper 17 cell differentiation via decreasing a disintegrin and metalloprotease 17 level in primary sjögren’s syndrome

**DOI:** 10.1080/21655979.2020.1870321

**Published:** 2021-01-15

**Authors:** Xiaoyan Wang, Shaojun Xin, Youqing Wang, Duo Ju, Quan Wu, Ye Qiu, Xuemin Niu, Wei Liu, Jianyou Li, Pengtian Ji

**Affiliations:** a Clinical Laboratory, Huzhou Central Hospital, Affiliated Central Hospital Huzhou University, Huzhou, China; b Department of Interventional Radiology, Huzhou Central Hospital, Affiliated Central Hospital Huzhou University, Huzhou, China; c Department of Orthopedics, Huzhou Central Hospital, Affiliated Central Hospital Huzhou University, Huzhou, China

**Keywords:** Primary sjögren’s syndrome, th17 cell differentiation, mir-146a-5p, adam17, il-23/IL-23R signaling

## Abstract

In clinical practice, we found that microRNA (miR)-146a-5p is significantly up-regulated in peripheral blood mononuclear cells (PBMCs) of primary sjögren’s syndrome (pSS) patients. *In vitro* experiments confirmed that miR-146a-5p promotes T helper 17 (Th17) cell differentiation, but the specific mechanism is still unknown. To solve this problem, 20 pSS patients and 20 healthy subjects were enrolled in this study and PBMCs were isolated from their blood. The expression of the membrane IL-23 R (mIL-23 R) in PBMCs was determined. CD3^+^ T cells were also isolated and used to further analyze the relationship between the ectodomain shedding of mIL-23 R and a disintegrin and metalloprotease 17 (ADAM17). Finally, miR-146a-5p inhibitor and mimics were transfected into PBMCs to evaluate the relationship between ADAM17 and mIL-23 R, and explore the role of mIL-23 R and ADAM17 in Th17 cell differentiation. Our results revealed a significantly increased expression of miR-146a-5p in PBMCs from pSS patients and significantly increased percentage of Th17 cells compared to PBMCs from healthy controls. Under polarization culture conditions, pSS patient-derived PBMCs can more easily differentiate into Th17 cells, which was, to a great extent, attributable to the increased expression of mIL-23 R. Moreover, ADAM17, an ectodomain sheddase of mIL-23 R, was targeted and negatively regulated by miR-146a-5p, which reduced the ectodomain shedding of mIL-23 R. Overall, our results suggested that miR-146a-5p could promote Th17 cell differentiation through targeting and negatively regulating ADAM17. Thus, these results might offer a new approach in the treatment of pSS.

## Graphical abstract

**Figure:**
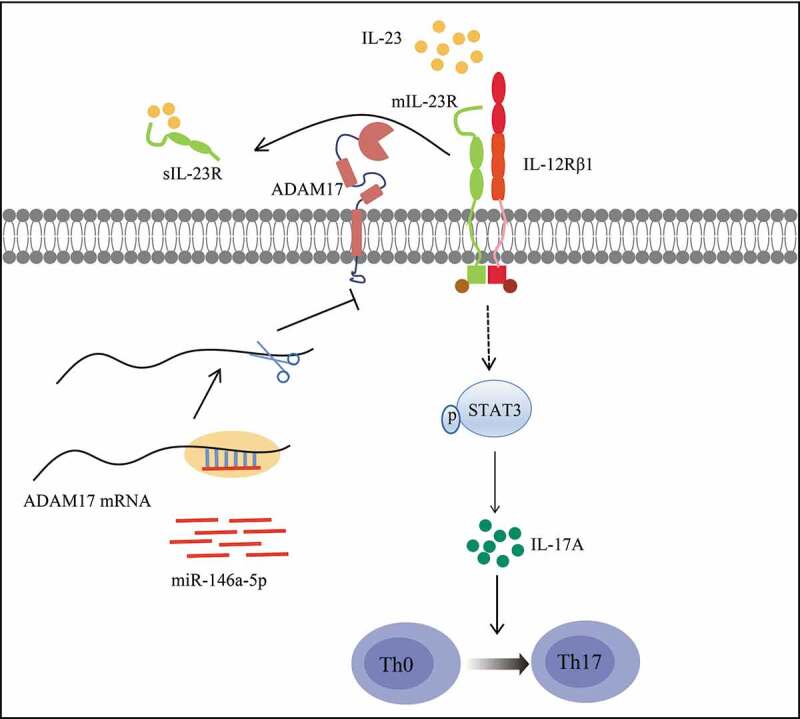

## 1. Introduction

Primary sjögren’s syndrome (pSS) is a systemic autoimmune disease mainly characterized by a chronic inflammation of the exocrine glands [1]. The progressive lymphocytic infiltration of salivary and lacrimal glands produces anti-SS-A/Ro antibody, anti-SS-B/La antibody, or other exocrine gland-specific antibodies, resulting in an impaired secretion function of the exocrine glands [2]. Increasing evidence suggests that T cells play a critical role in the systemic dysfunction of the immune response and the occurrence and development of tissue damage in pSS patients [3,4]. In particular, according to the recent discovery that Th17 cells are IL-17-secreting T cells, there is a growing evidence that Th17 cells instead of regulatory T (Treg) cells serve as the main pathogenic effector, inducing inflammation and autoimmunity in pSS glands, further causing gland damage [5–8].

**Figure 1.**
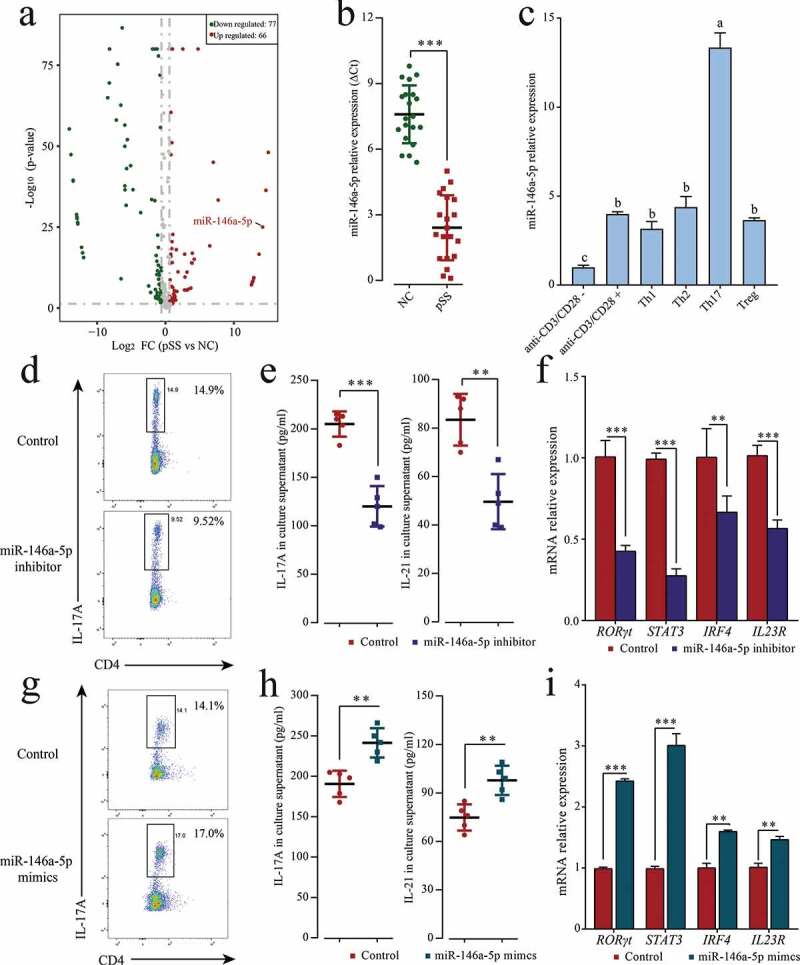
MiR-146a-5p up-regulated in pSS patients positively regulates Th17 cell differentiation. (a) Differentially expressed miRNAs in pSS patient-derived PBMCs displayed by miRNA chips. Red denotes up-regulated miRNAs (*p* ≤ 0.05, FC ≥ 2); green denotes down-regulated miRNAs (*p* ≤ 0.05, FC ≤ 2); gray denotes the absence of significant difference. (b) miR-146a-5p expression in patient-derived and NC-derived PBMCs by RT-qPCR. (c) Induced differentiation of pSS patient-derived PBMCs into different T cell subsets *in vitro*, and measurement of miR-146a-5p expression in different T cell subsets by RT-qPCR. ‘anti-CD3/CD28-’ means induction in the absence of CD3/CD28 antibody and cytokines; ‘anti-CD3/CD28+’ means induction in the presence of CD3/CD28 antibody only and in the absence of cytokines. Under *in vitro* Th17 polarization conditions, pSS patient-derived PBMCs were transfected with miR-146a-5p inhibitor or mimics. (d, g) Changes in the percentage of Th17 cells after the transfection of the inhibitor or mimics by flow cytometry. (e, h) IL17A and IL-21 level in cell medium supernatant after the transfection of the inhibitor or mimics by ELISA. (f, i) Expression of Th17-related transcription factors RORγt, STAT3, and IRF4 and cell surface receptor IL23R mRNA after transfection of the inhibitor or mimics by RT-qPCR. ** *p* ≤ 0.01, *** *p* ≤ 0.001 by Student’s *t*-tests

In recent years, the epigenetic regulation of the differentiation of T cell subsets has become a research hotspot, particularly, the regulation driven by microRNAs (miRNAs). As a class of single-chain, non-coding RNAs widely distributed in eukaryotes, miRNAs are usually 22–25 nucleotides long. By binding with the 3ʹ-untranslated regions (3ʹ-UTRs) of target genes, miRNAs can degrade mRNAs or inhibit mRNA translation, thus regulating the expression of target genes. MiRNAs also take part in the epigenetic modifications of a series of biological processes, including the differentiation of T cell subsets in the immune response [9–13]. MicroRNA (miR)-146a-5p is located on chromosome 5 and is a member of the miR-146 family. Numerous evidence demonstrated that the dysregulation of miR-146a-5p is associated to systemic lupus erythematosus (SLE) [14,15], rheumatoid arthritis (RA) [16,17], autoimmune thyroid disorder (AITD) [18,19], Graves’ disease (GD) [20,21], and many other autoimmune diseases. Some studies [22–24] discovered an increased expression of miR-146a-5p in the peripheral blood or peripheral blood mononuclear cells (PBMCs) of pSS patients. Sun et al. [25] proposed the use of miR-146a-5p as a biomarker of pSS based on meta-analysis. Our preliminary experiments revealed that miR-146a-5p is highly expressed particularly in Th17 cells, and positively regulates Th17 cell differentiation (Figure 1). However, the specific mechanism used by miR-146a-5p to promote Th17 cell differentiation in pSS is still to be clarified.

The differentiation and proliferation of Th17 cells rely on the cytokine IL-23, which is a member of the IL-12 family and a heterodimeric cytokine consisting of the p40 subunit and a unique p19 subunit of IL-12 [26]. By binding its membrane receptor mIL-23 R, IL-23 promotes the phosphorylation of STAT3, which subsequently induces the differentiation, maturation, and proliferation of Th17 cells [27,28]. Some studies also indicated that STAT3 can directly bind the promoters of IL-17 [29] and IL-21 [30], thus regulating the expression of interleukins. Studies that used immunodeficient mouse models found that the lack of IL-23 (*p19*) or IL-23 R (*Il23ra*) blocks the secretion of IL-17 [31,32]. Antibody therapies in clinical practice targeting IL-23 p19 are effective in treating psoriasis [33]. IL-23 R deficient cells are resistant to Th17-mediated autoimmune diseases, such as SLE [34]. These studies testify the vital role of the IL-23/IL-23 R signaling pathway in Th17 cell differentiation.

A recent study identified the surface IL-23 R as a substrate for a disintegrin and metalloprotease 17 (ADAM17). ADAM17 is a membrane-anchored enzyme that sheds the ectodomains of the nearby membrane-bound proteins into soluble proteins [35]. For example, it sheds IL-23 R ectodomain into soluble IL-23 R (sIL-23 R) by acting on IL-23 R domain 1 and domain 3, thus blocking IL-23/IL-23 R signaling [36]. In addition, ADAM17 exerts an uncoupling effect on more than 20 substrates, including cytokines, cytokine receptors, cell adhesion molecules, and chemokine receptors, all representing important immune molecules [37].

According to these loads of evidence, our hypothesis is that miR-146a-5p might affect the IL23/IL23R signaling pathway and further regulate Th17 cell differentiation via ADAM17 in pSS. To demonstrate this assumption, the expression of miR-146a-5p in pSS was evaluated in this study, and it was found that miR-146a-5p was increased in pSS patients, resulting in Th17 cell differentiation. Our *in vitro* experiments on PBMCs isolated from pSS patients validated our assumption that miR-146a-5p reduces the ectodomain shedding of mIL-23 R through targeting ADAM17, so as to activate the IL23/IL23R signaling pathway and promote Th17 cell differentiation.

## 2. Materials and methods

### 2.1. Patients and samples

The 20 pSS patients (female, aged 41 ± 17) enrolled in this study were all first-visit patients hospitalized in the Inpatient Department of the Huzhou Central Hospital from November 2012 to August 2013. The diagnosis of these patients conformed to the *Revised International Classification Criteria for Sjgren’s Syndrome (2002)* [38] and the *American College of Rheumatology Classification Criteria for Sjgren’s Syndrome (2012)* [39]. A total of 20 healthy female volunteers were also recruited and used as normal controls (NC), who were similar to pSS patients in age (42 ± 11) and body mass index (BMI). Subjects with other autoimmune diseases, diabetes, hypertension, tumor, or infectious diseases were excluded from both groups. None of the subjects was under drug or hormone treatment that affects the immune system. This study was approved by the Institutional Review Board of the Huzhou Central Hospital. All subjects provided the informed consent prior to their enrollment.

Venous blood samples were collected from the subjects after overnight fasting, and plasma was separated and stored at −80°C for further use. PBMCs were isolated from fresh blood through Ficoll (Solarbio, China) density gradient centrifugation, and cryopreserved in liquid nitrogen.

### 2.2. RNA extraction

Larger RNAs and miRNAs were extracted from pSS patient-derived and NC-derived PBMCs and *in vitro* induced T cells using a Micro RNA Kit (Omega Bio-Tek, USA), and used for subsequent miRNA sequencing and quantitative verification, respectively.

### 2.3. miRNA microarray

Peripheral blood samples were collected from both pSS patients and NCs, followed by PMBC isolation and miRNA extraction. The expression of miRNAs was detected using a miRNA Microarray chip (Sanger miRBase Ver. 20.0, LC Sciences, China). The hybridization images collected with a laser scanner were subjected to digital transformation using an Array-Pro analyzer (v6.3). As regard data processing and analysis, the first step was to correct the background, and redundant point values and standard deviations were calculated. Next, original chip data were standardized using the LOWESS algorithm [40], and standardized chip data were analyzed by the chi-square test. Bonferroni correction was performed to correct test results to reduce errors in the chi-square test. Finally, p value ≤ 0.05 and fold change (FC) ≥ 2 were used as the criteria to screen differential miRNAs.

### 2.4. Real-time quantitative reverse transcription PCR (RT-qPCR)

For RT-qPCR analysis of miR-146a-5p, miRNA was reversely transcribed to cDNA using a One Step miR cDNA Synthesis Kit (HaiGene, China) based on the manufacturer’s instructions. After reverse transcription, miR-146a-5p expression levels were evaluated using the SYBR® Premix Ex Taq^TM^ II kit (Takara, Japan) on an ABI 7500 RT-PCR instrument (Applied Biosystems, USA) with the following program: 95°C for 2 min, 40 cycles of 95°C for 15 sec, and 60°C for 30 sec. For determination of the expression of mRNA, total larger RNA was reversely transcribed using a ReverTra Ace qPCR RT Kit (Takara, Japan), and then evaluated using the SYBR® Premix Ex Taq^TM^ II kit (Takara, Japan). U6 small nuclear RNA and GAPDH were used as the internal controls of the RT-qPCR of miRNAs and mRNAs, respectively. Primer sequences are listed in Table S1. The relative expression was calculated using ΔCt (target gene Ct – internal control gene Ct) or 2^−ΔΔCt^.

### 2.5. Induced in vitro differentiation of T cell subsets

The *in vitro* differentiation of T cell subsets was induced according to the described with minor modifications [12]. A 24-well plate was pre-coated with anti-CD3 Abs (10 μg/ml) and anti-CD28 Abs (1 μg/ml), and 1 × 10^5^ PBMCs were added in each well. Cells were cultured in RPMI1640 supplemented with 10% FBS (Gibco, USA) and incubated at 37°C under 5% CO_2_. As regard Th17 cell differentiation, the medium was supplemented with rhIL-6 (25 ng/ml), rhIL-23 (25 ng/ml), rhTGFβ1 (10 ng/ml), and rhIL-1β (10 ng/ml), as well as neutralizing antibodies anti-human IL-4 (1 μg/ml) and anti-human IFNγ (0.5 μg/ml). As regard Th1 cell differentiation, the medium was supplemented with rhIL-12 (5 ng/ml) and anti-human IL4 (1 μg/ml). As regard Th2 cell differentiation, the medium was supplemented with rhIL-4 (20 ng/ml) and anti-human IFNγ (1 μg/ml). As regard Treg cell differentiation, the medium was supplemented with rhTGFβ1 (1 ng/ml). Cytokines and antibodies were purchased from Biolegend. After 4 days of induced culture under different conditions, cells were harvested for subsequent experiments.

### 2.6. Transfection of miR-146a-5p inhibitor or mimics

Fresh PBMCs isolated from pSS patients were transfected with miR-146a-5p mimics (40 nM), or inhibitor (40 nM), and control (Table S1) using Lipofectamine 3000 (Invitrogen, USA) under serum-free medium, respectively. After 12 h, the above Th17 polarization medium was used for continuous culture, and cells were harvested after 72 h. The percentage of Th17 cells were measured using flow cytometry. RT-qPCR was performed to detect the expression of the transcription factors RORγt, STAT3 and IRF4 and cell surface receptor IL-23 R mRNA. Cell medium supernatant was collected and the amount of IL-17A and IL-21 was measured by ELISA.

### 2.7. Flow cytometry

Fresh PBMCs isolated from pSS patients were resuspended in phosphate buffered saline (PBS). After inducing the culture *in vitro*, cells were harvested by centrifugation, washed with PBS to remove the medium, and resuspended in PBS. Then, they were stained with CD4-fluorescein isothiocyanate (FITC) antibody (BD Biosciences, USA) for 30 min at room temperature in the dark, and fixed and permeabilized using a Fixation/Permeabilization Solution Kit (BD Biosciences, USA). As regard the detection of the T cell subsets Th1 and Th17, cells were further stained using IFN-γ-phycoerthryrin-Cyanine7 (PE-cy7) antibody and IL-17A-phycoerthryrin (PE) antibody (eBioscience, USA). Treg cells were stained according to flow cytometry: The cells were first stained with CD4-FITC antibody and CD25-allophycocyanin (APC) antibody (eBioscience, USA) for 30 min at room temperature in the dark, then fixed and permeabilized using Foxp3 Fixation/Permeabilization buffer (eBioscience, USA), and finally stained using Foxp3-PE antibody (eBioscience, USA).

To detect the expression of the phosphorylated STAT3 (pSTAT3), PBMCs were cultured overnight in X–VIVO^TM^ 15 medium (Gibco, USA) in the presence or absence of rhIL-23 (25 ng/ml) stimulation [41]. Cells were first harvested, then fixed and permeabilized with Fix Buffer I and Perm Buffer III (BD Biosciences, USA), and finally stained with CD4-FITC antibody (BD Biosciences, USA), CD8-APC antibody (BD Biosciences, USA), and pSTAT3 (Y705)-PE (eBioscience, USA) antibody.

As regard the analysis of the membrane surface proteins ADAM17 and mIL-23 R, cells were stained with ADAM17-PE antibody (R&D Systems, USA) and IL-23 R-PE antibody (R&D Systems, USA), respectively. A BD LSRFortessa flow cytometer (BD Biosciences, USA) was used to examine the percentage of cells and the fluorescence intensity of proteins in the stained cells, and the FlowJo v10 software was used for data analysis.

### 2.8. Cytokine determination using enzyme-linked immunosorbent assay (ELISA)

The amount of IL-17A, IL-21, and sIL-23 R in the plasma and cell medium supernatant was measured using human IL-17A (ab216167), IL-21 (ab119542), and IL-23 R (ab267657) ELISA kits, respectively. All ELISA kits were purchased from Abcam (Cambridge, U.K), and all experiments were performed according to the manufacturer’s instructions.

### 2.9. Western blot (WB)

Total proteins were extracted from PBMCs using RIPA lysis buffer (Solarbio, China), and protein concentration was determined using the Pierce™ BCA Protein Assay Kit (Thermo Scientific, USA). An equal amount of protein was loaded onto 10% SDS-PAGE and subjected to electrophoresis, then transferred onto a polyvinylidene fluoride (PVDF) membrane (Bio-Rad, USA). The membrane was washed with Tris-buffered saline-Tween (TBST) (Solarbio, China), and blocked with 5% skim milk powder or bovine serum albumin (BSA) (Sigma-Aldrich, Germany). Incubation was carried out using specific primary antibodies against STAT3 (Abcam, U.K), pSTAT3 Y705 (Abcam, U.K), ADAM17 (R&D Systems, USA), IL-23 R (Abcam, U.K), and GAPDH (Santa Cruz, USA), followed by the incubation with the secondary antibodies HRP Goat anti-Mouse antibody (abclonal, USA) or HRP Goat Anti-Rabbit IgG (abclonal, USA). Pierce™ ECL Western Blotting Substrate (Thermo Fisher, USA) was used as the chemiluminescent substrate, and X-ray imaging was used to image the bands. Relative expression levels of proteins were quantified by densitometric values of fluorogram bands which was analyzed by Image J software (NIH, USA) and each value was normalized to those corresponding control protein band.

### 2.10. Luciferase assay

The binding site between ADAM17 and miR-146a-5p was predicted using the miRanda v3.3a software, and the 3ʹ-UTR (1691 bp, ENST00000310823.3) sequence of ADAM17 cloned into pGL3-Basic vector (Promega, USA). pGL3 ADAM17 3ʹUTR, pGL3 empty vector, miR-146a-5p mimics (at different doses of 20 nM and 40 nM), and negative controls were transfected into 293 T cells using Lipofectamine 2000 (Invitrogen, USA). pRL-TK vector was also transfected as the internal control reporter. Cells were harvested 24 h after transfection, and luciferase activity was detected using a Dual-Luciferase Reporter Assay System (Promega, USA) and a Lucetta Luminometer (Lonza, Germany). First, 10 μl cell lysis buffer was mixed with 10 μl luciferase assay reagent for the detection of firefly luciferase; then 10 μl Stop & Glo substrate was added for the detection of Renilla luciferase; finally, the ratio of firefly luciferase activity to Renilla luciferase activity was used to standardize the comparison in fluorescence intensity.

### 2.11. ADAM17 inhibition experiment

CD3^+^ T cells were isolated from PBMCs using a MojoSort^TM^ Human CD3 T Cell Isolation Kit (BioLegend, USA). The purity of isolated CD3^+^ T cells was above 95%, as examined by flow cytometry. ADAM17 inhibition experiment was carried out according to previous report [41]. Isolated CD3^+^ T cells were cultured in X–VIVOTM 15 medium containing 20% FBS, and treated for 1 h in the presence or absence of TAPI-1 (20 mM). Next, they were incubated on a plate coated with anti-CD3 antibody (10 μg/ml) and anti-CD28 antibody (1 μg/ml) for 4 h. Cells were harvested after treatment, and flow cytometry was performed to measure the fluorescence intensity of ADAM17 and IL-23 R in CD4^+^ T cells.

### 2.12. Statistical analysis

Statistical analysis was performed using the SPSS 22.0 software (SPSS lnc., USA). Results were expressed as mean ± standard deviation (M± SD), and intergroup comparison was carried out using Student’s *t*-tests or one-way analysis of variance (ANOVA). Correlations between variables were determined with Pearson correlation coefficients. A value of *p* < 0.05 was considered statistically significant.

## 3. Results

***3.1. MiR-146a-5p expression is increased in pSS patients, and positively regulates Th17 cell differentiation***

To comprehensively understand epigenetic regulation role of miRNA in pSS patients, miRNA chip was used to analyze differentially expressed miRNAs. A total of 143 miRNAs were obtained from pSS patient-derived and NC-derived PBMCs (pSS patients *vs*. NCs), divided into 66 up-regulated miRNAs and 77 down-regulated miRNAs (Figure 1a, Table S2). As further confirmed by RT-qPCR, miR-146a-5p was up-regulated by 18,853 times (Figure 1b). To further investigate the expression characteristics of miR-146a-5p in cells, the differentiation of PBMCs derived from pSS patients was induced *in vitro* into different T cell subsets such as Th1, Th2, Th17, and Treg. The expression of miR-146a-5p in these T cell subsets was measured using RT-qPCR and the results showed that miR-146a-5p was highly expressed in Th17 cells, but low expressed in Th1, Th2, and Treg cells (Figure 1c). To verify whether the specific high expression of miR-146a-5p in Th17 cells was due to Th17 cell differentiation, miR-146a-5p inhibitor and mimics were transfected into pSS patient-derived PBMCs and the cells were cultured under *in vitro* Th17 polarization conditions. MiR-146a-5p inhibitor clearly reduced the percentage of Th17 cells (Figure 1d), and down-regulated the mRNA expression of Th17 cell-related cytokines IL-17A and IL-21 (figure 1f), transcription factors RORγt, STAT3, and IRF4, and cell surface receptor IL23R (Figure 1 h). On the contrary, the transfection of miR-146a-5p mimics led to an increased percentage of Th17 cells, as well as the expression of cytokines, transcription factors, and cell surface receptor (Figure 1e, G, I). These results suggested that miR-146a-5p expression was increased in pSS patients, and positively regulated Th17 cell differentiation.

### 3.2. Th17 cells enhanced differentiation and increased proportions in pSS patients

**Figure 2.**
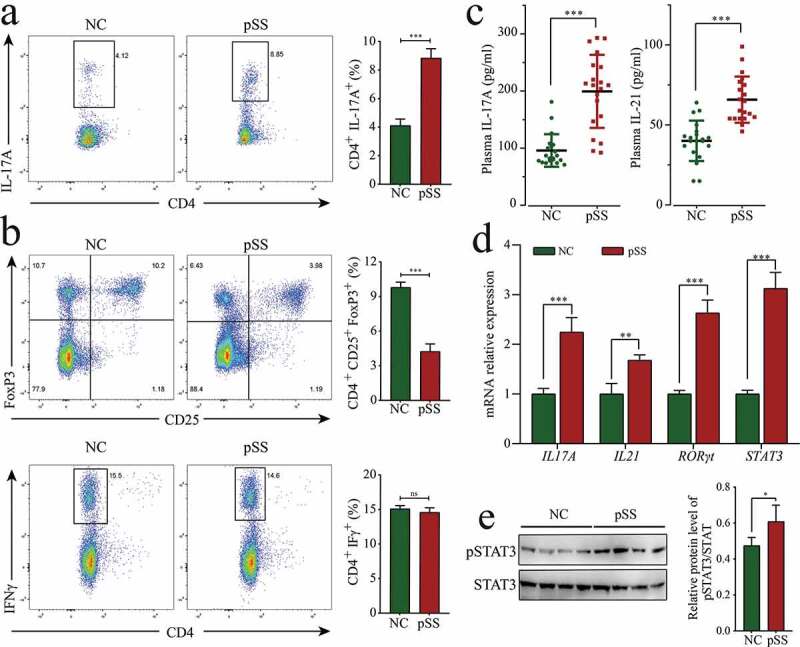
The increase in the percentage of Th17 cells in pSS patients. (a) Changes in the percentage of Th17 (CD4^+^ IL-17A^+^) in the PBMCs of pSS patients and NCs. (b) Changes in the proportions of Treg (CD4^+^ CD25^+^ FoxP3^+^) and Th1 (CD4^+^ IFN-γ^+^) in the PBMCs of pSS patients and NCs. (c) Levels of IL-21 and IL-17A in the plasma of subjects by ELISA. (d) MRNA expression of the cytokines IL-21 and IL-17A and transcription factors RORγt and STAT3 in the PBMCs of subjects by RT-qPCR. (e) pSTAT3 (Y705) protein expression in the PBMCs of subjects by WB. *ns*: not significant, ** *p* ≤ 0.01, *** *p* ≤ 0.001 by Student’s *t*-tests

Since the high expression of miR-146a-5p in pSS positively regulated Th17 cell differentiation, we wondered if the percentage of Th17 cells could be also increased as expected in pSS. To solve this doubt, the percentage of T cell subsets in the PBMCs of pSS patients was measured using flow cytometry. PSS patients showed an increase in the percentage of Th17 cells (CD4^+^ IL17^+^) by 4.63% (*p* < 0.0001), a decline in the proportions of Treg cells (CD4^+^ CD25^+^ FoxP3^+^) by 6.12% (*p* < 0.0001), and no obvious change in the proportions of Th1 cells (CD4^+^ IFN-γ^+^) compared to NCs (Figure 2a, B). PBMCs were cultured *in vitro* under Th17 polarization and non-polarization conditions for 72 h. The results showed that the percentage of Th17 cells in pSS patients was uniformly higher than that in NCs, and the increase in Th17 cells was more evident under polarization conditions in pSS patients (Figure S2). Besides, the expression of Th17 cell-related cytokines and transcription factors was detected using ELISA and RT-qPCR. The results indicated that the level of IL-17A in the plasma of pSS patients was significantly up-regulated by 2.08 times (*p* < 0.0001), and IL-17A mRNA expression in PBMCs was significantly up-regulated by 2.25 times (*p* < 0.0001) (Figure 2c, D). The level of IL-21 in plasma and IL-21 mRNA expression in PBMCs were significantly up-regulated by 1.64 (*p* < 0.0001) and 1.68 times (*p* = 0.0068), respectively. The expression of the Th17-related typical transcription factors RORγt and STAT3 in pSS patient-derived PBMCs was significantly up-regulated by 2.63 and 3.12 times (*p* < 0.0001, respectively) (Figure 2d). No evident changes were detected in the mRNA expression of Th1-related typical cytokine IFNγ or IL18, or Th2-related typical cytokine IL4 or IL13 (Figure S1). To further confirm the high percentage of Th17 cells in pSS, the expression of pSTAT3 was evaluated by WB, and the results revealed that its expression was higher (FC = 1.28, *p* = 0.039) in pSS patient-derived PBMCs than that in NCs (Figure 2e, S3A). To sum up, pSS patients showed enhanced differentiation and increased percentage of Th17 cells compared to NCs.

### 3.3. IL-23 significantly up-regulates pSTAT3 expression in pSS patient-derived PBMCs, and its increase is related to the expression of IL-23 R

**Figure 3.**
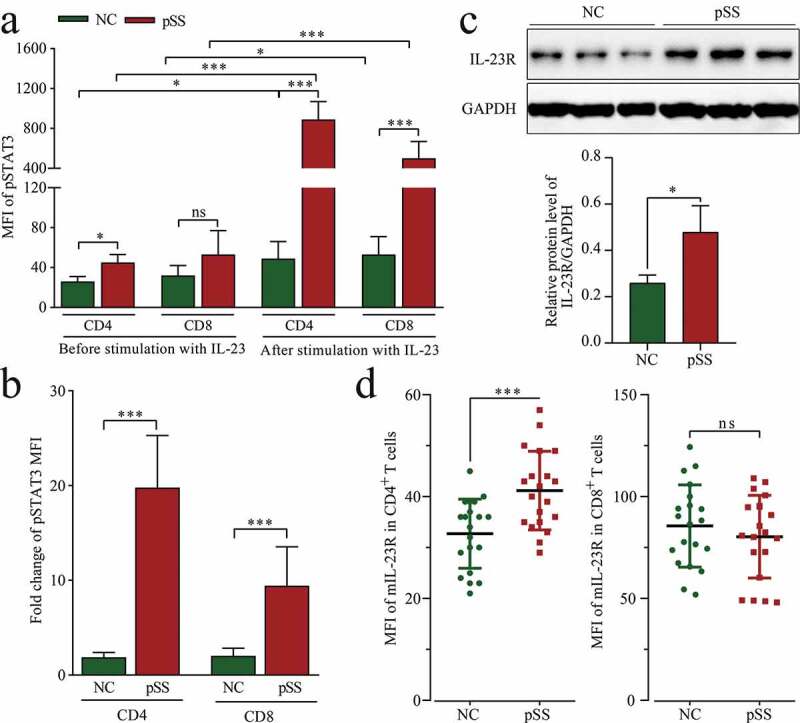
IL-23 significantly increases pSTAT3 expression in pSS patient-derived PBMCs, depending on the expression of IL-23 R: (a) MFI of pSTAT3 (Y705) in CD4^+^ and CD8^+^ T cells before and after IL-23 stimulation of PBMCs by flow cytometry. (b) FC of the MFI of pSTAT3 (Y705) before and after IL-23 stimulation. (c) IL-23 R protein expression in PBMCs by WB. (d, e) MFI of mIL-23 R in CD4^+^ and CD8^+^ T cells by flow cytometry. *ns*: not significant, * *p* ≤ 0.05, ** *p* ≤ 0.05, *** *p* ≤ 0.001 by ANOVA or Student’s *t*-test

The phosphorylation of STAT3 represents the progress of Th17 cell differentiation, and embodies the response to IL-23 stimulation. This, we next wondered if the enhanced Th17 cell differentiation in pSS was due to a higher sensitivity to IL-23 stimulation? To answer this question, pSS patient-derived and NC-derived PBMCs were tested *in vitro* in the presence or absence of IL-23 stimulation. According to the results, the mean fluorescence intensity (MFI) of pSTAT3 in CD4^+^ T cells in the absence of IL-23 stimulation was higher in pSS patients than in NCs; no significant difference was observed between the two groups in the expression of pSTAT3 in CD8^+^ T cells. After IL-23 stimulation, pSTAT3 expression in both CD4^+^ T and CD8^+^ T cells was significantly higher in pSS patients than in NCs (Figure 3a). In addition, as a result of IL-23 stimulation, the MFI levels of pSTAT3 in CD4^+^ T cells were increased several times, and significantly higher than those in CD8^+^ T cells. The MFI of pSTAT3 in CD4^+^ T cells was increased by a higher rate in pSS patients than in NCs (Figure 3b). These results indicated that pSS patient-derived CD4^+^ T cells produced hypersensitivity to IL-23 stimulation.

The vital role of IL-23/IL-23 R in Th17 cell differentiation and the hypersensitivity of pSS patient-derived CD4^+^ T cells to IL-23 stimulation stimulated our interest in the expression of IL-23 R. As shown in Figure 3c and S3B, the expression of IL-23 R was up-regulated (FC = 1.84, *p* = 0.023) in pSS patient-derived PBMCs, and such up-regulation occurred only in CD4^+^ T cells, but not in CD8^+^ T cells (Figure 3d). On the basis of the above results, it can be concluded that the hypersensitivity of pSS to IL-23 stimulation was attributable to an increased expression of IL-23 R.

### 3.4. MiR-146a-5p targets and regulates ADAM17

The regulatory effect of miR-146a-5p on Th17 cell differentiation was discovered thanks to the above results, but the potential regulatory mechanism still needs to be clarified. Analysis by miRanda revealed that miR-146a-5p could target and bind ADAM17 at 1,527–1,546 nt with Energy: −17 kcal/Mol. Manuel et al. [36] reported that ADAM17 blocks the IL23-STAT3 signaling pathway by acting on IL-23 R. Then, we wondered of miR-146a-5p could exert its regulatory effect through targeting ADAM17 in pSS patients.

**Figure 4.**
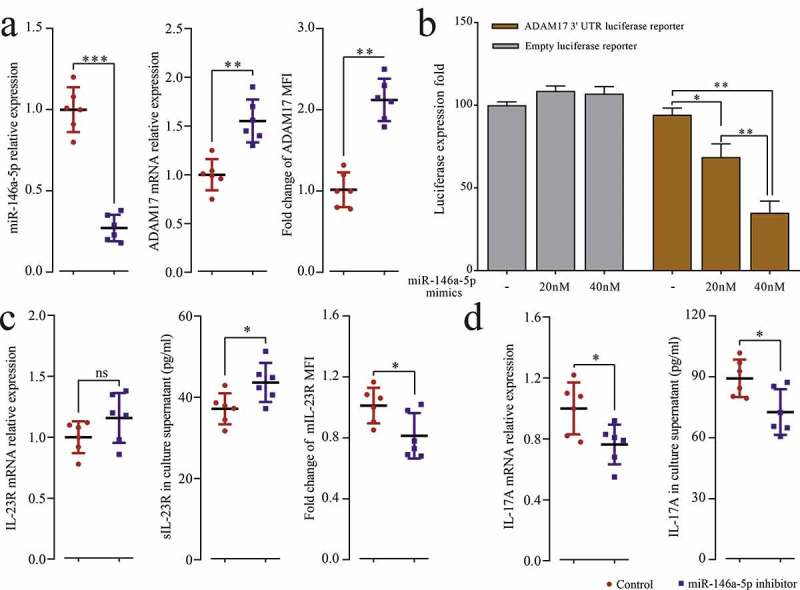
MiR-146a-5p targets ADAM17. PBMCs isolated from pSS patients and NCs were transfected with miR-146a-5p inhibitor and control. (a) miR-146a-5p expression and ADAM17 mRNA by RT-qPCR, and MFI of ADAM17 by flow cytometry. (b) ADAM17 protein expression by WB. (c) Transfection of pGL3 ADAM17 3ʹ UTR luciferase reporter vector or pGL3 empty vector and co-transfection of miR-146a-5p mimics at different doses in 293 T cells, and detection of MFI by Dual-Luciferase Reporter Assay System. PBMCs isolated from pSS patients and NCs were transfected with miR-146a-5p inhibitor and control. (d) IL-23 R mRNA expression by RT-qPCR, sIL-23 R level in the cell medium supernatant by ELISA, and MFI of mIL-23 R by flow cytometry. (e) IL-17A mRNA expression by RT-qPCR, and IL-17A level in the cell medium supernatant by ELISA. *ns*: not significant, * *p* ≤ 0.05, ** *p* ≤ 0.05, *** *p* ≤ 0.001 by Student’s *t*-tests

To explore the relationship between miR-146a-5p and ADAM17 in pSS patients, miR-146a-5p inhibitor was transfected into pSS patient-derived PBMCs, and the results showed that the inhibition of endogenous miR-146a-5p significantly up-regulated the protein and mRNA expression of ADAM17 (Figure 4a). pGL3 ADAM17 3ʹUTR vectors were constructed, and with the increase of the dose of miR-146a-5p mimics, the fluorescence intensity of the reporter gene drastically dropped by 25.67% (*p* = 0.0243) and 59.33% (*p* = 0.0011) compared to NCs (dose of mimics at 0). In contrast, no significant changes were observed in the empty vector group (Figure 4b). Moreover, after transfection of miR-146a-5p, the uncoupling of mIL-23 R increased (Figure 4c), accompanied by increased mRNA and protein expression of IL-17A (Figure 4d). Thus, the results suggested that miR-146a-5p could directly target and regulate ADAM17, further affecting the downstream IL23/IL-23 R signaling pathway and Th17 cell differentiation.

### 3.5. ADAM17 regulates the expression of mIL-23 R

**Figure 5.**
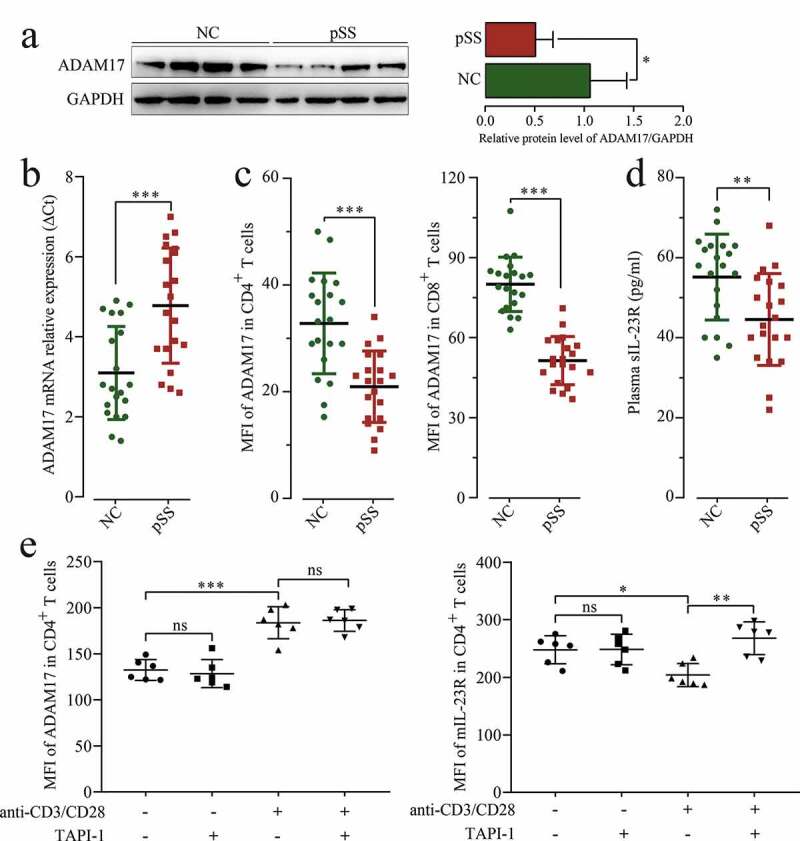
MIL-23 R serves as a substrate for ADAM17. (a) ADAM17 protein expression in pSS patient-derived and NC-derived PBMCs by WB. (b) ADAM17 mRNA expression in PBMCs by RT-qPCR. (c) MFI of ADAM17 in CD4^+^ and CD8^+^ T cells by flow cytometry. (d) sIL-23 R level in pSS patients and NCs plasma by ELISA. (e) In vitro culture of CD3 + T cells isolated from pSS patient-derived PBMCs with and without anti-CD3/CD28 stimulation in the presence or absence of TAPI-1, and MFI of ADAM17 and mIL-23 R in CD4^+^ T cells by flow cytometry. *ns*: not significant, * *p* ≤ 0.05, ** *p* ≤ 0.05, *** *p* ≤ 0.001 by ANOVA or Student’s t-test

To further verify that miR-146a-5p regulates IL23/IL23R signaling pathway through targeting ADAM17 in pSS patients, the expression of ADAM17 was detected in pSS patients. As shown in Figure 5a, S3C and B, the mRNA (ΔCt = 1.62, *p* < 0.001) and protein (FC = 0.48, *p* = 0.035) expression of ADAM17 was significantly down-regulated in pSS patients. PSS patients showed a down-regulation of ADAM17 in both CD4^+^ T and CD8^+^ T cells compared to NCs (Figure 5c). The level of sIL-23 R in the plasma was also detected using ELISA, and the results revealed that its level was significantly down-regulated in pSS patients than in NCs (Figure 5d), and a positive correlation was observed between the level of sIL-23 R and the MFI of AMAD17 in CD4^+^ T cells (r = 0.582, *p* < 0.001).

To confirm the uncoupling effect of ADAM17 on IL-23 R ectodomain, the purified CD3^+^ T cells isolated from pSS patients were treated with TAPI-1 (ADAM17 inhibitor), and ADAM17 and mIL-23 R percentage was measured by flow cytometry. As shown in Figure 5e, the expression of ADAM17 has increased under T cell receptor (TCR) activation; however, after TAPI-1 treatment, the uncoupling of mIL-23 R was inhibited, and the expression of mIL-23 R was increased. In summary, ADAM17 targeted and inhibited by miR-146a-5p in pSS patients promoted the expression of mIL-23 R, and further facilitated Th17 cell differentiation.

## 4. Discussion

In this study, a high expression of miR-146a-5p in pSS was found, and our results demonstrated that miR-146a-5p positively regulated Th17 cell differentiation. A total of 20 pSS patients and 20 healthy subjects were enrolled in this study, and the expression of mIL-23 R in PBMCs was detected at first. Then isolated CD3^+^ T cells were used to further analyze the relationship between the ectodomain shedding of mIL-23 R and ADAM17. Finally, miR-146a-5p inhibitor and mimics were transfected into PBMCs to verify the relationship between ADAM17 and mIL-23 R and explore the role of mIL-23 R and ADAM17 in Th17 cell differentiation.

Th17 cell differentiation and Treg cell differentiation share the same naïve CD4^+^ T cells and are both regulated by the TGF-β signaling pathway, but they play different roles. To be specific, Th17 cells recruit neutrophils at infection sites through the generation of IL-17, IL-22, and IL-23, and exert a pro-inflammatory effect. On the contrary, Treg cells inhibit the activity of multiple immunocytes through the generation of the anti-inflammatory cytokines IL-10 and TGF-β, and inhibit the immune response. In brief, Th17 cells induce autoimmunity, while Treg cells inhibit it and their balance plays a vital role in autoimmune diseases. In fact, increased Th17/Treg ratios are reported in many autoimmune diseases, such as RA [42], multiple sclerosis (MS) [43], inflammatory bowel disease (IBD) [44], and pSS [45]. Th17/Treg balance is affected by a series of factors, such as complex cytokine environments. In particular, normally TGF-β induces the differentiation of naïve CD4^+^ T cells into Treg cells; however, in the presence of IL-6 and IL-21, TGF-β induces the differentiation of naïve CD4^+^ T cells into Th 17 cells [46,47]. Th17 cells can also activate the secretion of IL-21. In this study, a high IL-21 expression was detected in pSS patients, which matched with the high percentage of Th17 cells. Furthermore, IL-6 and IL21 can activate STAT3, which further coordinates with IRF4 in inducing the expression of the transcription factor RORγt, thus promoting the differentiation of T cells into Th17 cell subsets [48,49]. In this study, a high expression of STAT3, RORγt, and other Th17-related transcription factors was also found in pSS patients. One limit of our study is that the expression of Treg-related factors was not explored. This may be the focus of our follow-up research on Th17/Treg balance in pSS.

Many existing studies reported the abnormal expression of miR-146a-5p in autoimmune diseases [14–22]. In this study, a high expression of miR-146a-5p was found in pSS, which was consistent with previous reports [22–24]. When CD4^+^ T cells isolated from pSS patients were transfected with miR-146a-5p inhibitor, the percentage of Th17 cells was reduced. On the contrary, when they were transfected with the mimics, the percentage of Th17 cells increased. These results demonstrated the vital regulatory effect of miR-146a-5p on Th17 cell differentiation in pSS. In this study, it was speculated that ADAM17 served as the target gene of miR-146a-5p according to miRanda, and this speculation was proved by the reporter gene assay. Indeed, our results showed that ADAM17 protein expression was reduced in pSS, and it was up-regulated by the transfection of miR inhibitor. These results suggested that high miR-146a-5p expression took part in the pathogenesis of pSS through targeting ADAM17.

A critical signal transduction role in autoimmune diseases is played by IL-23/IL-23 R, especially in terms of inducing Th17 cell differentiation. Some studies showed that sIL-23 R can act as an extracellular receptor analog by competitively binding IL-23, and inhibit Th17 cell differentiation through the inhibition of STAT3 signaling pathway [50,51,52]. sIL-23 R derives from different transcripts produced by alternative splicing [53] or the posttranslational modification of proteins. According to a recent study, the shedding of mIL-23 R ectodomain under the uncoupling effect of ADAM17 can also generate sIL-23 R [36]. In this study, the expression of sIL-23 R was lower in pSS patients than in NCs, but the expression of mIL-23 R was higher in the former than in the latter. Taking into account the low expression of ADAM17 in pSS, our hypothesis was that high expression of sIL-23 R in NCs was partially resulted from the uncoupling effect of ADAM17 on mIL-23 R. After the transfection of miR-146a-5p inhibitor, the expression of ADAM17 was increased. The examination of IL-23 R showed a significantly down-regulated mIL-23 R expression and significantly up-regulated sIL-23 R expression, which further confirmed the uncoupling effect of ADAM17 on mIL-23 R. The low ADAM17 expression increased mIL-23 R expression in pSS, which might explain why pSS was more sensitive (by increasing the percentage of Th17 cells) to polarization induction (IL-23 stimulation) *in vitro*.

## 5. Conclusions

Generally, ADAM17 can make mIL-23 R ectodomain into sIL-23 R through acting on IL-23 R, thus blocking IL-23/IL-23 R signaling. But in pSS patients, we found that elevated miR-146a-5p can activate IL-23/IL-23 R signaling via down-regulating expression level of ADAM17, therefore enhancing Th17 cell differentiation. Together, miRNA-146a-5p can enhance Th17 cell differentiation via deceasing ADAM17 level in pSS.

## Supplementary Material

Supplemental Material

## Highlights

Increased miR-146a-5p decreases ADAM17 level in pSS patient.

Decreased ADAM17 activates IL-23/IL-23R signaling.

Elevated miR-146a-5p promotes Th17 cell differentiation via targeting ADAM17.
