# Supplementary figures and images for: MicroRNA-146a-5p enhances T helper 17 cell differentiation via decreasing a disintegrin and metalloprotease 17 level in primary sjögren’s syndrome
Source: Bioengineered. 2021 Jan 15;12(1):310–24. doi: 10.1080/21655979.2020.1870321 (PMC8806215; doi:10.1080/21655979.2020.1870321)

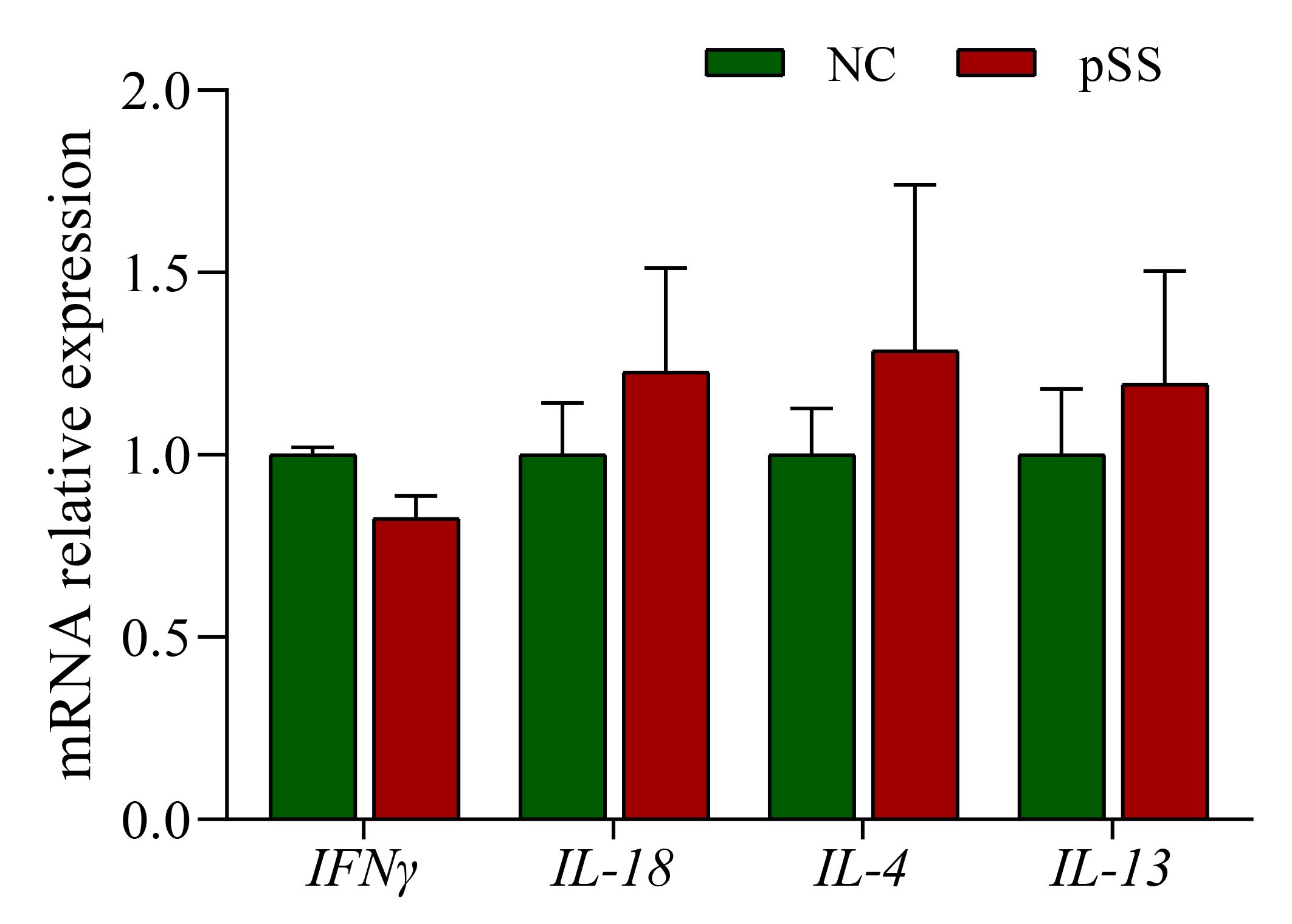

Supplement: Supplemental Material [file KBIE_A_1870321_SM7210.zip › SUPPLEMENTARY/Figure S1.tif]

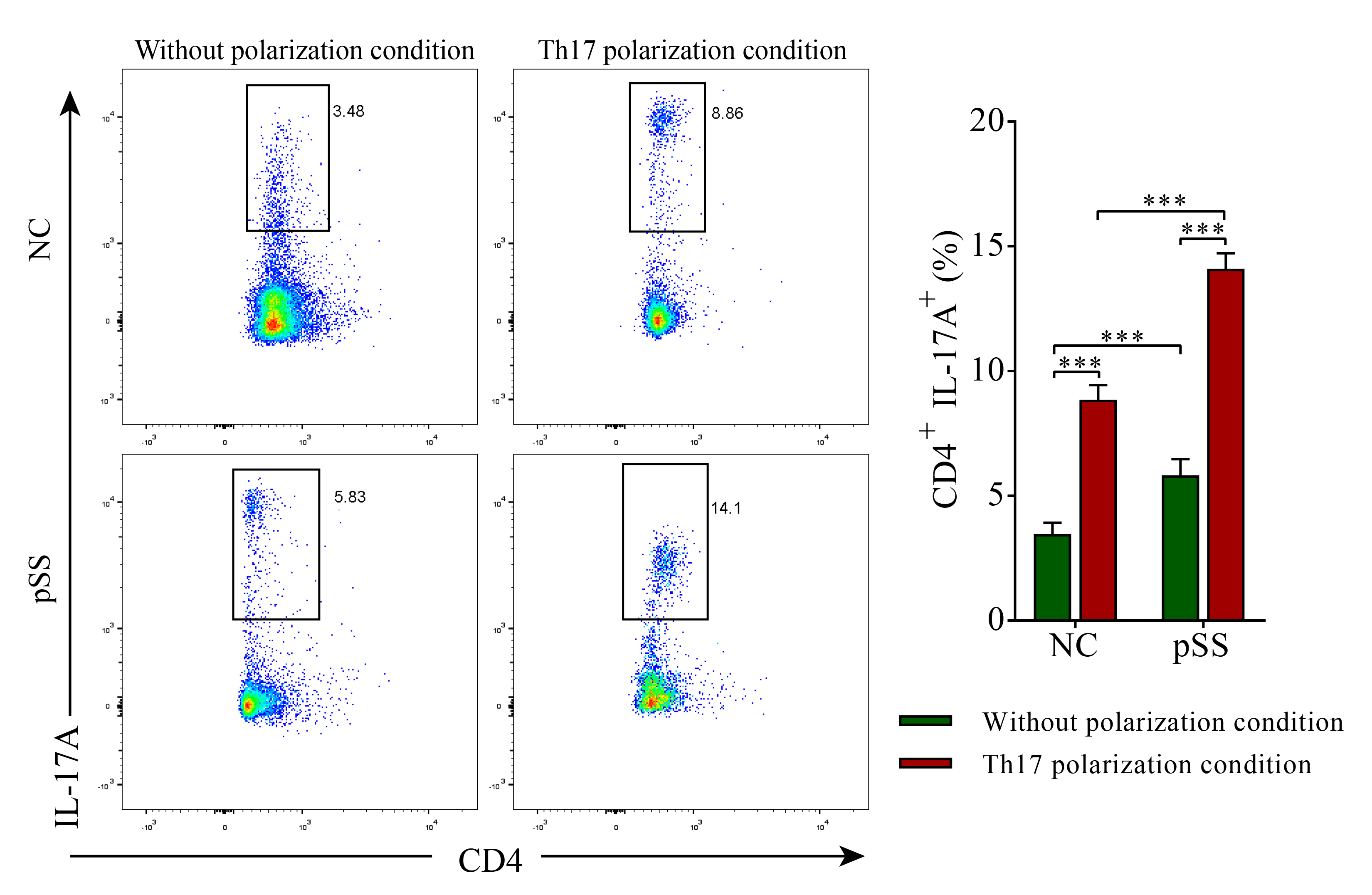

Supplement: Supplemental Material [file KBIE_A_1870321_SM7210.zip › SUPPLEMENTARY/Figure S2.tif]

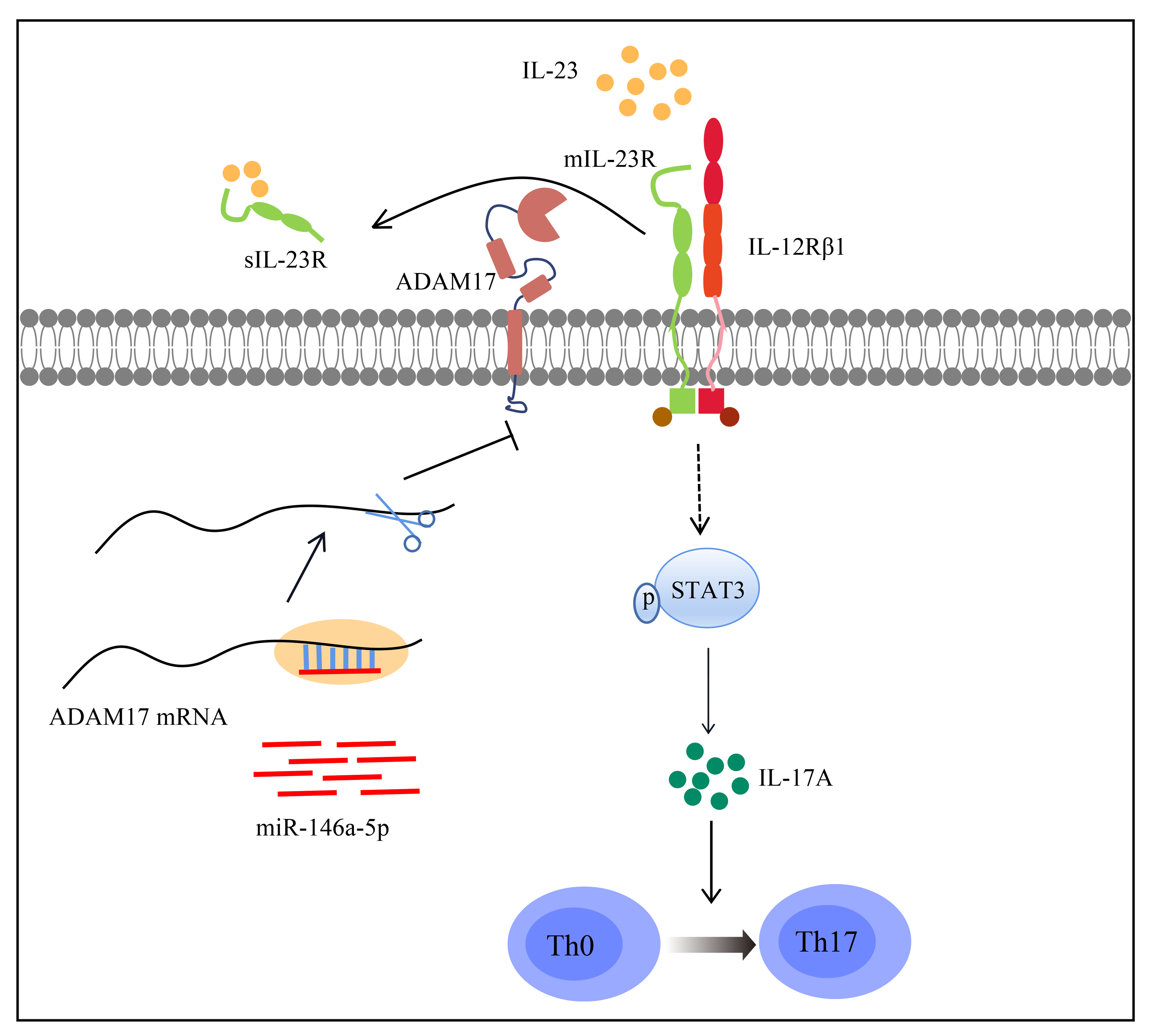

Supplement: Supplemental Material [file KBIE_A_1870321_SM7210.zip › SUPPLEMENTARY/Graphical Abstract.tif]
